# Warmer temperature and provision of natural substrate enable earlier metamorphosis in the critically endangered Baw Baw frog

**DOI:** 10.1093/conphys/coaa030

**Published:** 2020-06-17

**Authors:** Deon J Gilbert, Michael J L Magrath, Phillip G Byrne

**Affiliations:** 1 Wildlife Conservation and Science, Zoos Victoria, Elliott Avenue, Parkville, Victoria 3052, Australia; 2 School of BioScience, University of Melbourne, Victoria 3010, Australia; 3 School of Earth, Atmospheric and Life Sciences, Faculty of Science Medicine and Health, University of Wollongong, Wollongong, New South Wales 2522, Australia

**Keywords:** Development, food availability, growth, phenotypic plasticity, temperature, Wilbur–Collins

## Abstract

Temperature and food availability are known to independently trigger phenotypic change in ectotherms, but the interactive effects between these factors have rarely been considered. This study investigates the independent and interactive effects of water temperature and food availability on larval growth and development of the critically endangered Baw Baw frog, *Philoria frosti*. Larvae were reared at low (12°C) or high (17°C) water temperature in the absence or presence of substrate that controlled food availability, and body size and time to metamorphosis were quantified. Growth and development of larvae was influenced by the individual effects of temperature and food availability; time to metamorphosis was shorter in warm water treatment groups and in the presence of substrate and increased food. Unexpectedly, however, water temperature and food availability did not have an interactive effect on either time to metamorphose or body size at metamorphosis. Under all treatment groups, metamorphic onset occurred once a developmental size threshold was reached, indicating that growth rate and body size are key factors controlling the metamorphic process in Baw Baw frogs (consistent with the Wilbur–Collins model for ectotherm development). From an applied perspective, our findings have implications for amphibian conservation because they indicate that simple manipulations of temperature and food availability can be used to increase the rate of frog production in conservation breeding programs.

## Teaser

Optimizing captive growth and development has the potential to significantly improve the productivity and success of conservation breeding programs for threatened species. Here we investigate the independent and interactive effects of water temperature and food availability on larval growth and development of the critically endangered Baw Baw frog, *Philoria frosti*.

## Introduction

During ontogeny, individuals of many species respond to environmental changes by altering their phenotype so as to maximize their probability of survival and reproductive success (Sultan, 1995; Jannot, 2009; Tejedo *et al.*, 2010). This capacity for a single genotype to produce multiple phenotypes in response to different environments is termed phenotypic plasticity (Pigliucci, 2005). While many organismal traits are known to be plastic, three traits with particularly high levels of plasticity are somatic growth, development (ontogenetic change) and body size (Atkinson, 1994; Pigliucci, 2005). Among ectotherms, plasticity in growth, development and body size is particularly pronounced and is often attributed to variation in environmental temperature (Angilletta *et al.*, 2004). The ‘temperature size rule’ (TSR) predicts that ectotherms developing at colder temperatures will have a slower growth rate, a longer larval duration and a larger body size at metamorphosis (Atkinson, 1994). This rule has been extensively tested under controlled laboratory conditions, and the general finding is that ectotherms adhere to the TSR (Angilletta *et al*., 2004; Walters and Hassall, 2006). More broadly, the TSR is also supported by the finding that within species, populations found in colder environments typically have larger body size than those found in warmer regions (known as Bergman’s rule) (Bergmann, 1848). Moreover, in a review of empirical studies spanning bacterium, protists, plants and animals, Atkinson (1994) reported that 83.5% of 97 species (109 studies) showed decreased developmental size with increased temperature, lending further support to the TSR.

In addition to temperature, plasticity in ectotherm growth and development has also been linked to variation in food availability. Food availability is a factor often related to an organism’s ability to achieve maximal growth and reach metamorphic onset at a faster developmental rate (Jones *et al.*, 2016; Vaissi and Sharifi, 2016; Wolnicki *et al.*, 2016). Several theoretical models have considered how variation in food availability might affect organismal growth and development, but the most widely recognized is the Wilbur–Collins model (Wilbur and Collins, 1973). This model proposes that growth rate and body size are key factors controlling the metamorphic process and assumes that a minimum size threshold must be met before metamorphic onset. Importantly, Wilbur and Collins (1973) hypothesized a potential negative relationship between growth and development. They argued that larval development may progress more rapidly under periods of poor food availability as an adaptive response to enable individuals to escape a deteriorating environment, resulting in decreased growth and a smaller body size at metamorphosis. Many empirical studies have tested the validity of the Wilbur–Collins model for ectotherm growth and development, and while a diversity of species does not respond in the predicted way (Fenaux *et al.*, 1994; Takahashi and Watanabe, 2005; Rosen *et al.*, 2014), some species do (Alford *et al.*, 1988; Twombly, 1996; Beck and Congdon, 2000; Morey and Reznick, 2000). For example, Morey and Reznick (2000) demonstrated a negative relationship between growth and metamorphosis of three species of spadefoot toad (genus, *Scaphiopus)*. In their study, all three toad species displayed a plastic response to changes in food availability. Larvae experiencing low food availability developed faster and metamorphosed earlier than those experiencing high food availability, provided a minimum size threshold was reached (Morey and Reznick, 2000). This plastic response may improve prospects of survival to a new life stage but could have negative and long lasting consequences for lifetime reproductive success (Morey and Reznick, 2000). In contrast to the Wilbur–Collins model, the ‘metabolic down-regulation model’ predicts an overall metabolic depression as a result of decreased food availability, limiting processes such as growth and development (Keys *et al.*, 1950; Guppy and Withers, 1999). Overall, there is considerable support for the metabolic down-regulation model, with studies in a diversity of taxonomic groups finding that food deprivation results in metabolic depression and retarded growth and development (Fenaux *et al*., 1994; Guppy and Withers, 1999; Takahashi and Watanabe, 2005). In addition, there is also experimental evidence that these plastic responses can extend the time taken for individuals to reach metamorphosis (Jones *et al*., 2016, see also McLeod *et al.*, 2013; Vaissi and Sharifi, 2016).

While the effects of temperature and food availability on ectotherm growth and development have been examined in isolation (i.e. studies have tested the independent effects of these factors), there remains a limited understanding of how interactions between temperature and food availability shape life history traits. In principle, temperature might influence food availability in freshwater aquatic systems in two main ways. First, because of the positive relationship between temperature and decomposition rates, food availability might decrease at warmer temperatures, which might restrict rates of growth and development (Ferreira *et al.*, 2011). Second, because warmer temperatures result in higher metabolic rates and require organisms to increase food consumption to meet basic physiological requirements, rapid growth might only be experienced under conditions of adequate food availability at warmer temperatures (Hailey *et al.*, 2007; Jones *et al*., 2016).

To date, only a small number of experimental studies with fish and amphibians have attempted to explore the interactive effects of temperature and food availability on vertebrate growth and development, but the emerging pattern is that interactive effects are important. However, the direction of the relationships appears to vary considerably. Some studies have reported that, high food availability is required for optimal growth and development at high rearing temperatures (Mcleod *et al*., 2013; Vaissi and Sharifi 2016), while others report that high food availability is not essential (Newman, 1988), or may even be detrimental, particularly in regard to individual survival (Jones *et al*., 2016). This variation may reflect differences in experimental approaches (e.g. the temperate range tested), but it may also indicate species specific differences linked to life history variation. Clearly, additional studies are required to advance our understanding of how temperature and food availability interact to influence plasticity in ectotherm growth and development.

**Picture 1:**
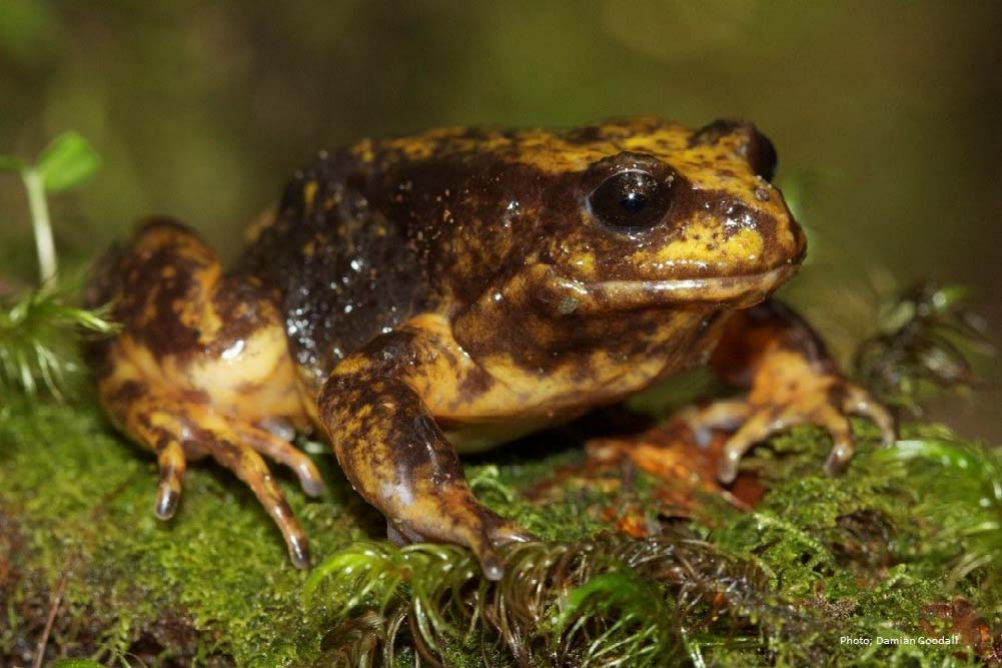
Adult male Baw Baw frog (*Philoria frosti*) in situ at Mt Baw Baw.

A better understanding of how temperature and food availability interact to influence plasticity in growth, development and body size will advance our understanding of factors controlling trait variation within and between populations and also assist with investigations concerning the evolution of phenotypic plasticity. Moreover, knowledge in this area may be of value to amphibian conservation (Canessa *et al.*, 2014; Sharifi *et al.*, 2014; Jones *et al*., 2016), particularly in terms of reintroduction where post release survival is often low (Dodd Jr *et al.*, 1991; Griffiths *et al.*, 2008). Amphibians are declining faster than any other vertebrate group, with recent International Union for Conservation of Nature (IUCN) data suggesting a staggering 53% of evaluated species currently at risk of extinction (IUCN, 2019). In response, a key management action has been the establishment conservation breeding programs (CBP’s) for endangered species (Stuart *et al.*, 2004; Browne *et al.*, 2011). Determining optimal temperature and nutritional requirements, and how they interact, will be critical for identifying rearing conditions that maximize the number of individuals that can be produced (Canessa *et al*., 2014; Tarszisz *et al.*, 2014) without compromising body size at metamorphosis, which is known to have major impacts on individual performance, both in captivity (Beck and Congdon, 2000; Hopwood *et al.*, 2014) and post release (Canessa *et al*., 2014). Within the captive environment, food availability for larval amphibians will be directly influenced by how much food is delivered per day, but it might also be impacted by the presence of substrate. Substrate type is an important abiotic factor known to influence food availability for developing aquatic larvae (Martínez-Cárdenas and Flores-Nava, 2004; Akers *et al.*, 2008; Hanlon *et al.*, 2015). Furthermore, empirical studies have demonstrated that substrate composition, particle surface area and organic load are important factors influencing colonization by zooplankton and phytoplankton communities and may play an important role in feeding efficiencies and larval survival (Hailey *et al*., 2007; Akers *et al*., 2008; Hanlon *et al*., 2015). For example, Akers *et al.* (2008) attributed increased larval growth of Cope’s gray tree frog *(Hyla chrysoscelis)* to meiofauna sequestered on the vast, collective surface area provided by clay substrates. Larval amphibians typically feed on algae and periphyton that grow on substrate, so providing substrate could significantly increase food availability. Furthermore, because primary productivity of these food sources is likely to increase with temperature, raising tadpoles on substrate at warmer temperatures may expedite their growth and development.

The Baw Baw frog, *Philoria frosti* is confined to an area of 135 km^2^ of the Mt Baw Baw plateau in Victoria, Australia (Hollis, 2004) and is restricted to protected montane gully habitat between 1000 m and 1300 m. Since the 1980s, wild populations have declined by up to 98%, and the species is now listed as critically endangered by the IUCN (www.iucnredlist.org). In 2010, the species became the focus of an intensive conservation breeding program, with the specific goal of securing a viable captive assurance population. With this goal now achieved, the focus has turned towards optimizing captive breeding protocols. To this end, the aim of our study was to assess the independent and interactive effects of water temperature and substrate on *P.frosti* larval growth and development. We reared larvae at low (12°C) and high (17°C) ecologically relevant water temperatures, and in the presence or absence of substrate and quantified effects on larval growth, developmental rate and body size at metamorphosis. We predicted significant interactions between temperature and substrate.

## Methods

### Study species

*Philoria frosti* is a medium-sized stout terrestrial frog with a large parotoid gland on each shoulder (Picture 1). Breeding habitat relies on wet soak and seepage lines underneath vegetation, fallen logs and rocks and breeding occurs annually. Egg masses are deposited underground and can be laid at varying depths, depending on the structural components of the site (Hollis, 2004). Oviposition sites are typically muddy and rich in organic matter with complex granular granite structure comprising of large rock to fine particles. Egg masses contain between 50–185 eggs (Littlejohn, 1963; Malone, 1985a) and are white and unpigmented. Hatching begins from 10 days after oviposition (Anstis, 2013) and may last for up to 5 days (D. Gilbert, pers. obs.). Larvae develop within the wet deposition site, though can be free swimming if washed in to nearby streams (Malone, 1985a; Hollis, 2004). Under natural conditions, development to metamorphosis takes 10–18 weeks (Malone, 1985b). *P. frosti* does not follow normal Gosner (Gosner, 1960) stage development from stage 18 onwards (Anstis, 2013). Larval tooth rows are not present (Anstis, 2013) and larvae retain a large residual egg yolk that is capable of sustaining development through to metamorphosis (Littlejohn, 1963). However, behaviour observed during captive rearing suggests larvae may feed on egg mass jelly, unfertilized or dead eggs and/or deceased tadpoles (D Gilbert, pers. obs.).

### Study animals

Three fertilized *P.frosti* egg masses (containing 80, 65 and 95 ggs, respectively) were collected from Mt Baw Baw on the 22nd of November 2015. Clutches were most likely laid by different females, but two clutches may have had the same sire as they were removed from one nest site. The date of egg deposition was unknown; however, all three clutches appeared to be at a similar stage of development. Egg masses were individually packed into 5 L aquarium bags and partially filled (~1 L) with water collected from oviposition site. Each bag was placed into a 1 L food container (Sistema Pty Ltd), all air was expelled and the lid was sealed with duct tape to prevent water loss. Each container was packed into a small Styrofoam cooler box (30 cm × 30 cm × 20 cm) with two frozen ice bricks to maintain internal temperature of 10°C. Egg masses were transported in an air-conditioned car to Melbourne Zoo on the 23rd of November 2015. Each of the three egg masses was placed in a separate plastic rearing tub (40 cm × 40 cm × 8 cm) with ~2 cm of reverse osmosis (RO) water filtering through to waste. All tadpoles had hatched after 26 days and were moved to experimental treatment containers at 5 days after hatching. A total of 72 individuals (*n* = 24 from each clutch) were separated into individual rearing containers (10.5 cm × 10 cm × 10 cm) and raised until metamorphosis (complete tail absorption) under one of four experimental treatments (see section 2.4 below).

### Experimental design

**Figure 1:**
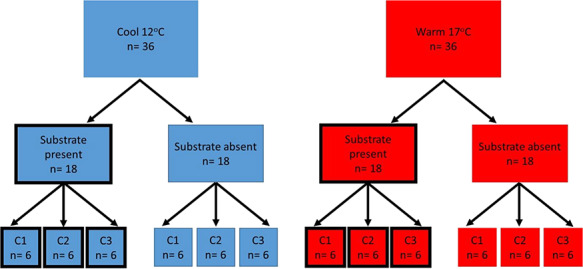
Diagram showing the experimental design used to test the influence of temperature and substrate on the larval development of *P.frosti*. Larvae were reared under one of two temperatures (cold or hot) and two substrate availability conditions (substrate present or substrate absent). C denotes clutch number within each treatment group.

To test the individual and interactive effects of water temperature and substrate (food availability) on the growth and development of larvae, 18 individuals (6 from each of the 3 clutches collected) were allocated to each of the following four treatments: (i) ‘warm water (17°C) with substrate available’, (ii) ‘warm water with substrate unavailable’, (iii) ‘cool water (12°C) with substrate available’ and (iv) ‘cool water with substrate unavailable’ (Fig. 1). The cool water temperature (12°C) was selected to replicate oviposition site temperature recorded in montane habitat during egg collection*,* while the warm water temperature (17°C) was chosen as the maximum recorded temperature at oviposition sites in the historical frost hollow habitat (Malone, 1985a). The provision of substrate was the only possible food source during the experimental period (i.e. tadpoles were not provided with any other food source). Response variables measured were: (i) body size at weeks 1, 2, 3, 4, 5 and metamorphosis (total length mm—see below for methodology), (ii) time to metamorphosis (days) and (iii) weight at metamorphosis (g). The study concluded once all larvae had metamorphosed (complete tail absorption).

### Developmental husbandry

Tadpoles were housed in a constant temperature room (set at 12°C) in individual round plastic containers (Genfac 10 cm × 10 cm) filled with 600 ml of RO water. Individuals in the ‘substrate available’ treatments had 1 cm of substrate (decomposing granite and organic material) from the field oviposition site on the bottom of their rearing container. Groups of containers (*n* = 6) were submersed to within 1 cm of the top in water baths (*n* = 12), with substrate available in half of the containers (*n* = 3) (Fig. 1.). Cool water treatments were controlled using ambient room temperature. Warm water treatments were heated using small aquarium heaters (Marina Submersible Pre-Set Heater 15 cm 25w). Water baths alternated between cool and warm baths along the length of the facility to account for any spatial variation in room temperature. Due to the shelving configuration within the facility, six water baths were positioned 20 cm higher than the other six. Half the water in each housing container was changed three times per week. Water changes for containers in the warm treatment were made with water at the same temperature. Each tadpole was photographed weekly using an Olympus Tough digital camera for the duration of the experiment. A 10 mm calibration plastic square at the bottom of each container allowed body size measurements to the nearest 0.01 mm to be taken from these images using Image J (https://imagej.nih.gov/ij/). Weight at metamorphosis to the nearest 0.01 g was taken using digital scales (Pesola touch screen digital pocket scale). Larval development of the individuals was assessed daily. Once the first front limb was exposed, water depth in the container was dropped to 1 cm and a small (2 cm × 2 cm × 1 cm) piece of aquarium filter foam added to prevent drowning. Following complete metamorphosis, frogs were placed on a small (2.5 cm diameter) petri dish, weighed and photographed on top of a 1 cm^2^ scale bar.

### Statistical analysis

Generalized linear models (GLM’s) were used to test for the influence of temperature and substrate on larval growth and development. These models used a normal distribution and an identity link function. In each model, the explanatory variables were temperature and substrate (including an interaction term between temperature and substrate) and the response variables were either body size (mm) at weeks 1, 2, 3, 4 and 5, body size at metamorphosis (mm), weight at metamorphosis (g) or time to metamorphosis (days). The test statistics used for all GLMs were maximum likelihood Chi-squared tests (McCullagh, 2018). For each GLM model, post-hoc treatment comparisons were made between treatment groups using Wilcoxon Each Pair tests (McCullagh, 2018).

## Results

### Effect of temperature and substrate on tadpole body size

**Figure 2:**
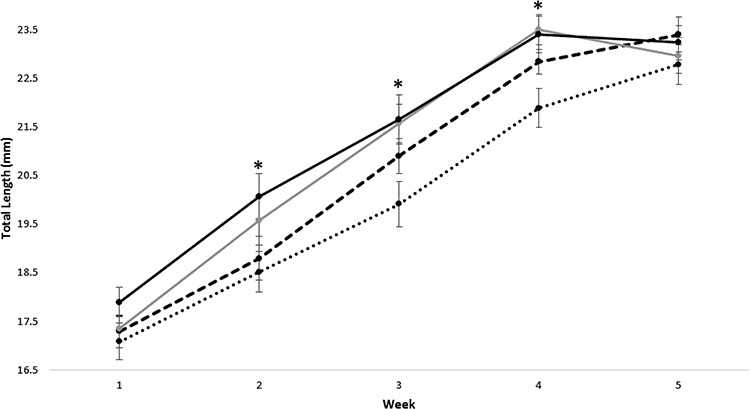
Effect of temperature and substrate on mean total length (mm) of larval *P. frosti* at week 1, week 2, week, 3, week 4 and week 5. The four treatment groups were (i) individuals raised in cool water with substrate unavailable (represented by dot line), (ii) individuals raised in cool water with substrate available (represented by dash line), (iii) individuals raised in warm water with substrate unavailable (represented by solid grey line) and (iv) individuals raised in warm water with substrate present (represented by solid black line). * denotes weeks where total length was significantly different between a least two of the treatment groups (*P* < 0.05).

Across the four treatment groups, tadpole body length increased between weeks 1 and 5, with mean body length ranging from 17.08 to 17.89 mm in week 1, 18.52 to 20.07 mm in week 2, 19.91 to 21.66 mm in week 3, 21.89 to 23.50 mm in week 4 and 22.79 to 23.41 mm in week 5 (see Fig. 2). In week 1, there was no significant effect of temperature (GLM, ${x}_1^2$= 1.888, *P* = 0.169) or substrate treatments (GLM, ${x}_1^2$= 1.436, *P* = 0.231) on body size nor was there a significant interaction between the temperature and substrate treatments (GLM, ${x}_1^2$= 0.273, *P* = 0.601). However, in weeks 2, 3 and 4, there was a significant effect of temperature treatment on body size (week 2: GLM, ${x}_1^2$= 6.456, *P* = 0.011; week 3: GLM, ${x}_1^2$= 7.679, *P* = 0.006; week 4: GLM, ${x}_1^2$= 10.011, *P* = 0.002), with tadpoles in the warmer treatments being larger than those in the cooler treatments (see Fig. 2). In contrast, there was no significant effect of substrate treatment on tadpole size over the same period (week 2: GLM, ${x}_1^2$= 0.756, *P* = 0.385; week 3: GLM, ${x}_1^2$= 1.582, *P* = 0.209; week 4: GLM, ${x}_1^2$= 1.694, *P* = 0.193) and no significant interaction between the temperature and substrate treatments (week 2: GLM, ${x}_1^2$= 0.063, *P* = 0.801; week 3: GLM, ${x}_1^2$= 1.135, *P* = 0.287; week 4: GLM, ${x}_1^2$= 2.482, *P* = 0.115). In week 5, there was no significant effect of temperature or substrate treatment on tadpole body size (temperature; GLM, ${x}_1^2$= 0.0004, *P* = 0.982; substrate; GLM, ${x}_1^2$= 1.492, *P* = 0.222). Nor was there a significant interaction between the temperature and substrate treatments (GLM, ${x}_1^2$= 0.238, *P* = 0.626).

### Effect of temperature and substrate on time to metamorphosis

**Figure 3:**
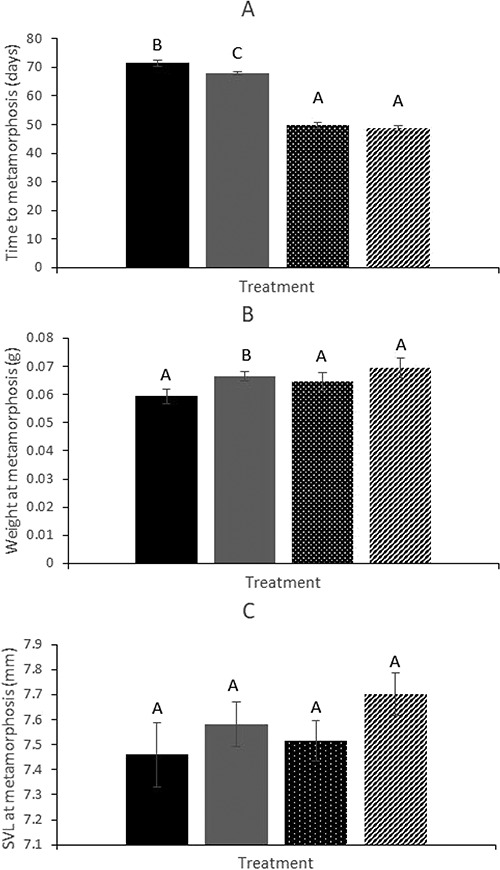
Effect of temperature and substrate on (**A**) time to metamorphosis (days), (**B**) weight at metamorphosis (g) and (**C**) snout-vent length at metamorphosis (mm). The four treatment groups were (i) individuals raised in cool water with substrate unavailable (represented by black bars), (ii) individuals raised in cool water with substrate available (represented by grey bars), (iii) individuals raised in warm water with substrate unavailable (represented by dot bars) and (iv) individuals raised in warm water with substrate present (represented by diagonal line bars). Treatments that share the same letter are not significantly different from each other, as determined by paired Wilcoxon tests.

Time to metamorphosis ranged from 48.7 to 71.4 days, and differed between treatment groups. Time to metamorphosis was shorter for tadpoles from warmer treatments (Fig. 3; GLM, ${x}_1^2$=143.765, P < 0.001). Furthermore, at both cool and warm temperatures, time to metamorphosis was shorter when substrate was present (Fig. 3; GLM, ${x}_1^2$= 6.129, P = 0.013). There was no significant interaction between temperature and substrate (GLM, ${x}_1^2$= 1.936, P = 0.164).

### Effect of temperature and substrate on frog size at metamorphosis

Body length at metamorphosis ranging from 7.46 mm to 7.70 mm and was similar across all four treatment groups (Fig. 3), with no significant effect of temperature (GLM, ${x}_1^2$= 0.850, *P* = 0.357) or substrate (GLM, ${x}_1^2$= 2.617, *P* = 0.106) on length at metamorphosis. There was also no significant interaction between temperature and substrate (GLM, ${x}_1^2$= 0.117, *P* = 0.733).

### Effect of temperature and substrate on frog weight at metamorphosis

Mean frog weight at metamorphosis varied from 7.46 mm to 7.70 mm across the four treatment groups (Fig. 3). This variation was partly explained by a significant effect of substrate (GLM, ${x}_1^2$= 4.442, *P* = 0.035), with treatments with substrate producing heavier frogs (Fig. 3.) However, there was no effect of temperature on weight at metamorphosis (GLM, ${x}_1^2$= 2.224, *P* = 0.136) nor was there a significant interaction between temperature and substrate treatments (GLM, ${x}_1^2$= 0.182, *P* = 0.670).

## Discussion

Food availability and temperature are known to trigger phenotypic change, but the interactive effects between these factors are only beginning to be considered. Here we investigate the individual and interactive effects of temperature and substrate availability on *P. frosti* larval growth and development. Tadpoles were reared under two different temperature treatments (cool 12°C and warm 17°C) and with substrate available or unavailable. Tadpole size increased between weeks 1 and 5, and there was a significant effect of temperature, but not substrate, on tadpole size in weeks 2–4. Moreover, tadpoles raised in the warmer water treatments reached metamorphosis significantly earlier than those raised under cool water treatments, regardless of the presence or absence of substrate. However, neither the temperature nor the substrate treatments affected the body size of frogs at metamorphosis. All tadpoles metamorphosed at ~7 mm in length, suggesting that *P. frosti* tadpoles must reach a threshold body size in order to metamorphose. Such developmental thresholds have been reported for other anuran species (Morey and Reznick, 2000; Day *et al.*, 2002; Lind *et al.*, 2008) and are thought to exist so that minimal physiological requirements are met before individuals transition into the adult life stage (Wilbur and Collins, 1973). Failure to meet a minimal threshold body size might result in developmental stasis or death if environmental conditions deteriorate before minimum developmental requirements are met (Morey and Reznick, 2000). This is particularly critical during early life when development is influenced by fluctuating biotic and abiotic factors such as temperature and food availability.

As predicted, the shorter time to metamorphosis for tadpoles from warmer treatments provides support for the notion that warmer temperature accelerates ectothermic development (Jones *et al*., 2016; Vaissi and Sharifi, 2016). Furthermore, the temperature we used for the warmer treatments (17°C) appears to be within the upper thermal limit for this species because survivorship to metamorphosis was high (94.5%). In addition, at both cool and warm water treatments, time to metamorphosis was marginally faster when substrate was present, suggesting some nutritional benefit to *P.frost*i associated with the presence of substrate. Indeed, past studies have shown that substrate can benefit tadpoles by increasing the availability of periphyton and algae, which is an important source of tadpole nutrition (Martínez-Cárdenas and Flores-Nava, 2004; Akers *et al*., 2008). However, it is important to note that *P.frosti* larvae were still able to reach metamorphic thresholds in the total absence of substrate, suggesting that embryonic yolk reserves in *P.frosti* are adequate to support growth and development in the complete absence of external food sources. Some other studies have demonstrated this mode of non-feeding development. For example, by raising three species of toad (genus, *Bufo*) individually and without food, Crump (1989) demonstrated that one species (*B. periglenes*) was capable of completing metamorphosis, while the remaining two species (*B. marinus*, and *B. coniferus*) suffered complete mortality. In nature, *P. frosti* typically develops underground in shallow water seeps or wet soak areas associated with decomposing pieces of granite rock or natural cavities created under vegetation (Littlejohn, 1963; Malone, 1985a; Hollis, 1995). Larvae typically stay within the oviposition site but have been recorded to swim small distances in shallow water (Hollis, personnal communication). Therefore, larger or more nutritious yolk reserves may be an adaptation that allows *P.frosti* to develop in a potentially nutrient poor environment (Crump, 1989) and reduce investment in morphological features or behaviours necessary for food acquisition or predation avoidance.

Our findings are in line with previous studies that have reported that food availability can underpin plasticity in larval growth and development in anurans (Álvarez *et al.*, 2002b; Jones *et al*., 2016). When considered in conjunction with size at metamorphosis, we conclude that *P. frosti* larvae raised under warmer treatments, in the presence of substrate, more rapidly reach the critical size threshold required for metamorphosis. However, it must be noted that our results only demonstrate outcomes for larval rearing to metamorphosis and do not assess post metamorphic fitness. Further investigation is required to determine outcomes on traits related to adult fitness, such as foraging performance and predator escape responses and/or whether warmer temperatures skew sex ratios, as has been reported for some amphibian species (Hayes, 1998; Dournon *et al.*, 2003). Importantly, the findings of this study do not support the TSR, which states that slower development results in larger size, particularly in organisms found in cold climates (Atkinson, 1994; Angilletta Jr. *et al.*, 2003; Atkinson *et al.*, 2006; Walters and Hassall, 2006). Instead, we found that larval *P.frosti* must meet minimum developmental thresholds for metamorphic onset, irrespective of developmental water temperature (at least within the range imposed in this study). These findings more closely align with predications of the Wilbur–Collins model that metamorphosis is determined when individuals reach a minimal size threshold and that onset of metamorphosis is determined by an individual’s growth history (Wilbur and Collins, 1973). Critical thresholds were more rapidly attained when larvae developed in the presence of substrate providing evidence that *P.frosti* have the ability to opportunistically feed on biotic growth when it is available at the oviposition site, despite the potential to reach metamorphosis in the complete absence of food. Because feeding in *P.frosti* is not essential to complete development (as discussed above), the implication is that individuals will feed opportunistically. Early life history of *P. frosti* is not characterized by prolonged larval development as seepage lines and wet soak depressions associated with oviposition sites are prone to drying. Moreover, such drying is reported to be a major cause of mortality under natural conditions (74.5% during embryonic development and 70.3% during the larval stage) (Malone, 1985a). Thermal variation between egg deposition sites may also offer an explanation why *P. frosti* requires developmental thresholds to initiate metamorphosis, instead of showing plasticity, in line with the TSR. Natural *P. frosti* oviposition sites have been recorded to show variation in water temperature of between 5°C and 17°C (Malone, 1985a). Our results showed earlier metamorphosis under warmer conditions, and therefore, decreased time to meet developmental thresholds for metamorphosis. This is consistent with the Wilbur–Collin prediction that there is a minimum size required for metamorphosis and that acceleration to this stage is appropriate under certain environmental conditions (Wilbur and Collins, 1973). Our results show *P. frosti* demonstrates developmental plasticity under different environmental conditions and indicate that minimal size thresholds are critical for metamorphic onset.

A number of recent studies have reported interactive effects of water temperature and food availability on ectotherm growth and development (McLeod *et al*., 2013; Jones *et al*., 2016; Vaissi and Sharifi, 2016). In contrast, we did not find any interaction between water temperature and food availability during *P. frosti* larval development. This may be due to a number of factors. In the substrate treatments, available food may not have decomposed significantly faster at the higher temperature. First, the temperature treatments may not have been different enough to result in significantly different levels of colonization and production of meiofauna communities. Second, available food in the substrate treatments may not have decomposed significantly faster at the higher temperature. Third, food may not have been limiting at the higher temperatures. Fourth, because substrate was the only source of food, food quality may not have been representative of the preferred food source for developing larvae. For example, in nature *P. frosti* larvae typically develop within the oviposition site (Hollis, 2004) and may feed on decomposing egg jelly, unfertilized eggs or dead larvae, as has been observed in captivity (D. Gilbert, pers. obs.).

## Conclusion

This study investigated the individual and interactive effects of temperature and substrate on the growth and development of larval *P. frosti.* Metamorphosis was found to be initiated by developmental body size thresholds, with a significant decrease in time to metamorphosis under warm water conditions. This provides support for the Wilbur–Collins model of ectotherm development (Wilbur and Collins, 1973). However, as larval rearing conditions are known to influence terrestrial life stage success (Álvarez *et al.*, 2002a), more work is needed to assess the long term effects of temperature and substrate treatments on post-metamorphic fitness-related traits, such as size at maturity, foraging performance and predator escape responses.

Importantly, our findings have implications for *P. frosti* conservation breeding because they indicate that simple manipulations of temperature and food availability can decreases time to metamorphosis, without compromising survival. This knowledge stands to increase the rate of frog production, which may have two major benefits. First, producing viable frogs more rapidly should reduce husbandry costs, which should improve our capacity to quickly build and maintain sustainable captive populations. Second, increasing the number of frogs available for release should improve reintroduction success, primarily by reducing the likelihood of genetic and demographic problems associated with small population sizes and/or densities (Fischer *et al.*, 2000; Armstrong *et al.*, 2008) More broadly, deepening our understanding of the independent and interactive effects of temperature and food availability has the potential to improve amphibian conservation breeding programmes globally.

## Funding

This study was funded by Zoos Victoria and supported by the School of Earth, Atmospheric and Life Sciences, University of Wollongong.

## Ethical approval

The protocols and procedures used in this research were reviewed and approved by the Zoos Victoria Animal Ethics Committee (ZV15078) in accordance with the National Health and Medical Research Council Australian code for the care and use of animals for scientific purposes.
